# Supplementary material for: Hedgehog signaling is a potent regulator of liver lipid metabolism and reveals a GLI-code associated with steatosis
Source: eLife. 2016 May 17;5:e13308. doi: 10.7554/eLife.13308 (PMC4869931; doi:10.7554/eLife.13308)
Supplement: Figure 1—source data 1. — DOI: http://dx.doi.org/10.7554/eLife.13308.004 [file elife-13308-fig1-data1.docx]

Figure 1 - source data 1

Source data of qRT-PCR of *Smo* in different tissues and isolated hepatocytes of the SLC-WT and the SLC-KO mice (Figure 1F).

| **figure** | **tissue** | **mean SLC-WT** | **SEM SLC WT** | **n** | **mean SLC-KO** | **SEM SLC-KO** | **p value**  **(t-test)** | **n** |
| --- | --- | --- | --- | --- | --- | --- | --- | --- |
| **1F** | heart-tissue | 1.00 | 0.48 | 7 | 2.23 | 1.11 | 0.2758 | 6 |
|  | lung-tissue | 1.00 | 0.35 | 6 | 1.23 | 0.51 | 0.7132 | 6 |
|  | spleen-tissue | 1.00 | 0.37 | 6 | 1.44 | 0.37 | 0.4124 | 6 |
|  | adipose-tissue | 1.00 | 0.47 | 8 | 0.42 | 0.18 | 0.3067 | 5 |
|  | liver-tissue | 1.00 | 0.35 | 19 | 0.05 | 0.001 | 0.0448* | 10 |
|  | hepatocytes | 1.00 | 0.28 | 10 | 0.09 | 0.03 | 0.0004*** | 16 |
