# Supplementary material for: Hedgehog signaling is a potent regulator of liver lipid metabolism and reveals a GLI-code associated with steatosis
Source: eLife. 2016 May 17;5:e13308. doi: 10.7554/eLife.13308 (PMC4869931; doi:10.7554/eLife.13308)
Supplement: Figure 2—source data 1. — DOI: http://dx.doi.org/10.7554/eLife.13308.007 [file elife-13308-fig2-data1.docx]

Figure 2 – source data 1

| **figure** | **analyzes** | **mean SLC-WT** | **SEM SLC WT** | **n** | **mean SLC-KO** | **SEM SLC-KO** | **p value**  **(t-test)** | **n** |
| --- | --- | --- | --- | --- | --- | --- | --- | --- |
| **2B** | fat red staining | 5.731 | 1.923 | 10 | 40.792 | 9.950 | 0.0009*** | 7 |

| **figure** | **analyzes** | **mean SLC-WT [%]** | **SEM SLC WT [%]** | **n** | **mean SLC-KO [%]** | **SEM SLC-KO [%]** | **p value**  **(t-test)** | **n** |
| --- | --- | --- | --- | --- | --- | --- | --- | --- |
| **2C** | liver/body ratio | 6.81 | 0.38 | 7 | 5.87 | 0.45 | 0.0139* | 7 |

Source data of quantification of the fat red-stained liver sections from the male SLC-WT and SLC-KO mice (Figure 2B) and comparison of the liver/body ratio (Figure 2C).
