# Supplementary material for: Hedgehog signaling is a potent regulator of liver lipid metabolism and reveals a GLI-code associated with steatosis
Source: eLife. 2016 May 17;5:e13308. doi: 10.7554/eLife.13308 (PMC4869931; doi:10.7554/eLife.13308)
Supplement: Figure 3—source data 1. — DOI: http://dx.doi.org/10.7554/eLife.13308.013 [file elife-13308-fig3-data1.docx]

| **figure** | **gene** | **mean SLC-WT** | **SEM SLC-WT** | **n** | **mean SLC-KO** | **SEM SLC-KO** | **p value**  **(t-test)** | **n** |
| --- | --- | --- | --- | --- | --- | --- | --- | --- |
| **3A** | *Ihh* | 1.00 | 0.14 | 6 | 0.62 | 0.10 | 0.050* | 6 |
|  | *Shh* | 1.00 | 0.12 | 7 | 0.57 | 0.12 | 0.028* | 8 |

Figure 3 – source data 1

| **figure** | **gene** | **mean SLC-WT** | **SEM SLC-WT** | **n** | **mean SLC-KO** | **SEM SLC-KO** | **p value**  **(t-test)** | **n** |
| --- | --- | --- | --- | --- | --- | --- | --- | --- |
| **3B** | *Ptch1* | 1.00 | 0.52 | 9 | 1.16 | 0.21 | 0.400 | 10 |
|  | *Ptch2* | 1.00 | 0.03 | 15 | 0.96 | 0.25 | 0.8958 | 13 |
|  | *Hhip* | 1.00 | 0.16 | 8 | 1.74 | 0.46 | 0.1499 | 8 |

| **figure** | **gene** | **mean SLC-WT** | **SEM SLC-WT** | **n** | **mean SLC-KO** | **SEM SLC-KO** | **p value**  **(t-test)** | **n** |
| --- | --- | --- | --- | --- | --- | --- | --- | --- |
| **3C** | *Fu* | 1.00 | 0.20 | 15 | 1.05 | 0.15 | 0.9456 | 15 |
|  | *Sufu* | 1.00 | 0.22 | 14 | 1.25 | 0.23 | 0.4175 | 15 |

| **figure** | **gene** | **mean SLC-WT** | **SEM SLC-WT** | **n** | **mean SLC-KO** | **SEM SLC-KO** | **p value**  **(t-test)** | **n** |
| --- | --- | --- | --- | --- | --- | --- | --- | --- |
| **3D** | *Gli1* | 1.00 | 0.23 | 13 | 0.33 | 0.06 | 0.0114* | 10 |
|  | *Gli2* | 1.00 | 0.24 | 9 | 0.93 | 0.34 | 0.8623 | 7 |
|  | *Gli3* | 1.00 | 0.17 | 13 | 0.47 | 0.13 | 0.0195* | 12 |

Source data of the expression of genes related to Hh signaling in SLC mice (Figure 3A-D).
