# Supplementary material for: Hedgehog signaling is a potent regulator of liver lipid metabolism and reveals a GLI-code associated with steatosis
Source: eLife. 2016 May 17;5:e13308. doi: 10.7554/eLife.13308 (PMC4869931; doi:10.7554/eLife.13308)
Supplement: Figure 4—source data 1. — DOI: http://dx.doi.org/10.7554/eLife.13308.017 [file elife-13308-fig4-data1.docx]

**Figure 4-source data 2**

**Gene set enrichment analysis of isolated hepatocytes from SLC-WT and SLC-KO mice.**

**up-regulated genes**

| **GO identifier** | **GO term** | **group size** | **hits in group** | **gene symbol** | **p-value** |
| --- | --- | --- | --- | --- | --- |
| GO:0006629 | lipid metabolic processes | 947 | 30 | *9130409I23Rik, Aacs, Acot2, Acot3, Adipor2, Akr1d1, Aldh3a2, Angptl4, Apob, B3galt1, Cd36, Cds2, Chpt1, Cnbp, Cyp17a1, Cyp51, Cyp7a1, Elovl6, Fasn, Gpam, Hmgcr, Ldlr, Lipg, Lpin1, Mtm1, Pltp, Ppara, Pten, St3gal5, Thrsp* | 7,70E-11 |
| GO:0008152 | metabolic processes | 9767 | 113 | *1810073K19Rik, 2810442I22Rik, 4833442J19Rik, 9130409I23Rik, Aacs, Aadacl1, Acot2, Acot3, Acss2, Adipor2, Akr1d1, Alas1, Aldh3a2, Alkbh5, Angptl4, Apob, Aqp11, Aqp4, Arfgap3, B3galt1, BC016495, Ccrn4l, Cd36, Cds2, Chpt1, Clpx, Cnbp, Csad, Cyp17a1, Cyp51, Cyp7a1, Dclre1a, Dct, Dnajb9, Dnase2a, Dpysl2, Ece1, Eef2k, Elovl6, Fasn, Fmo2, Gadd45a, Gck, Gda, Glul, Gmfb, Gpam, Gpt2, Gsta2, Gstm2, ...* | 1,24E-09 |
| GO:0006082 | organic acid metabolic processes | 731 | 20 | *Aacs, Acot2, Acot3, Adipor2, Akr1d1, Cd36, Csad, Cyp7a1, Dct, Elovl6, Fasn, Gck, Glul, Gpam, Khsrp, Me1, Ppara, Ppat, Prodh, Tpr* | 1,61E-06 |
| GO:0006694 | steroid biosynthetic processes | 123 | 7 | *Akr1d1, Apob, Cnbp, Cyp17a1, Cyp51, Cyp7a1, Hmgcr* | 6,09E-05 |
| GO:0009725 | response to hormone stimulus | 257 | 9 | *Adipor2, Ldlr, Lpin1, Me1, Ppara, Ramp2, Rcan1, Tgfbr2, Txnip* | 0,000234984 |
| GO:0050832 | defense response to fungus | 5 | 2 | *1810073K19Rik, Cotl1* | 0,000630691 |
| GO:0001569 | patterning of blood vessels | 24 | 3 | *Cyr61, Gna13, Tgfbr2* | 0,000907719 |
| GO:0009966 | regulation of signal transduction | 788 | 15 | *Aplp2, Arfgap3, Centb2, D0H4S114, Ece1, Farp2, Hspa1b, Lect1, Lgals1, Onecut1, Pten, Ramp2, Rgs16, Slc20a1, Zcchc11* | 0,00169518 |
| GO:0006878 | cellular copper ion homeostasis | 11 | 2 | *Aplp2, Cp* | 0,0033607 |
| GO:0006414 | translational elongation | 38 | 3 | *Eef2k, Gtpbp2, Trim24* | 0,00348546 |
| GO:0051346 | negative regulation of hydrolase activity | 38 | 3 | *Angptl4, Chpt1, Hspa1b* | 0,00348546 |
| GO:0015936 | coenzyme A metabolic processes | 12 | 2 | *Hmgcr, Pank3* | 0,00401166 |
| GO:0018105 | peptidyl-serine phosphorylation | 40 | 3 | *Map3k12, Morc3, Sbk1* | 0,00403466 |
| GO:0000160 | two-component signal transduction system (phosphor) | 43 | 3 | *Per1, Per2, Per3* | 0,0049522 |
| GO:0007179 | transforming growth factor beta receptor signaling | 91 | 4 | *D0H4S114, Nfic, Onecut1, Tgfbr2* | 0,0062187 |
| GO:0046148 | pigment biosynthetic processes | 48 | 3 | *Alas1, Dct, Ppat* | 0,00674127 |
| GO:0007584 | response to nutrient | 151 | 5 | *Adipor2, Cp, Hspa1b, Lipg, Tgfbr2* | 0,00748225 |
| GO:0008277 | regulation of G-protein coupled receptor protein s | 50 | 3 | *Ece1, Ramp2, Rgs16* | 0,0075509 |

**down-regulated genes**

| **GO identifier** | **GO term** | **group size** | **hits in group** | **gene symbol** | **p-value** |
| --- | --- | --- | --- | --- | --- |
| GO:0051258 | protein polymerization | 105 | 7 | *Ang, Pdgfc, Pfn2, Sco2, Sgk1, Tubb2a, Tubb5* | 0,00013635 |
| GO:0032147 | activation of protein kinase activity | 99 | 4 | *Ang, Gadd45g, Pdgfc, Prkcn* | 0,0015948 |
| GO:0007017 | microtubule-based processes | 338 | 7 | *100041586, Arcn1, Dnaic1, Dynlt1, Sgk1, Tubb2a, Tubb5* | 0,0016224 |
| GO:0009239 | enterobactin biosynthetic process | 49 | 3 | *BC089597, Rdh20, Rdh9* | 0,00194595 |
| GO:0048878 | chemical homeostasis | 560 | 9 | *Avpr1a, Cldn1, EG666577, Gclc, Hist1h1c, Saa1, Sco2, Sgk1, Stat3* | 0,00207999 |
| GO:0009314 | response to radiation | 188 | 5 | *Ccnd1, Cdkn1a, Dnmt3b, Hist1h1c, Sgk1* | 0,00265168 |
| GO:0051222 | positive regulation of protein transport | 55 | 3 | *Ang, Saa1, Smo* | 0,00271116 |
| GO:0048010 | vascular endothelial growth factor receptor signal | 16 | 2 | *Hhex, Nrp1* | 0,00289652 |
| GO:0008284 | positive regulation of cell proliferation | 397 | 7 | *Ang, Ccnd1, Cdkn1a, Crip2, Nrp1, Pdgfc, Smo* | 0,00397708 |
| GO:0051186 | cofactor metabolic processes | 298 | 6 | *BC089597, Gclc, Gss, Mcm7, Rdh20, Rdh9* | 0,00402025 |
| GO:0007049 | cell cycle | 1037 | 12 | *Ccnd1, Cdkn1a, Fbxo39, Gadd45g, Hhex, Mad2l2, Mcm7, Sgk1, Trp53inp1, Tubb2a, Tubb5, Zzef1* | 0,00588549 |
| GO:0009892 | negative regulation of metabolic processes | 704 | 9 | *Ang, Cdkn1a, Dnmt3b, Fst, Gclc, Hhex, Sgk1, Stat3, Tcf4* | 0,00916225 |
