# Supplementary material for: Hedgehog signaling is a potent regulator of liver lipid metabolism and reveals a GLI-code associated with steatosis
Source: eLife. 2016 May 17;5:e13308. doi: 10.7554/eLife.13308 (PMC4869931; doi:10.7554/eLife.13308)
Supplement: Figure 4—source data 2. — DOI: http://dx.doi.org/10.7554/eLife.13308.018 [file elife-13308-fig4-data2.docx]

Figure 4 – source data 1

| **figure** | **gene** | **mean SLC-WT** | **SEM SLC-WT** | **n** | **mean SLC-KO** | **SEM SLC-KO** | **p value**  **(t-test)** | **n** |
| --- | --- | --- | --- | --- | --- | --- | --- | --- |
| **4B** | *Chrebp1* | 1.00 | 0.23 | 6 | 2.61 | 0.56 | 0.0341* | 7 |
|  | *Srebf1* | 1.00 | 0.15 | 12 | 1.99 | 0.12 | < 0.0001*** | 12 |
|  | *Srebf2* | 1.00 | 0.18 | 15 | 2.13 | 0.14 | < 0.0001*** | 18 |
|  | *Ppara* | 1.00 | 0.16 | 15 | 7.60 | 0.49 | 0.0033** | 14 |
|  | *Pparab/d* | 1.00 | 0.25 | 9 | 2.16 | 0.60 | 0.1644 | 12 |
|  | *Pparg* | 1.00 | 0.30 | 8 | 2.37 | 0.50 | 0.034* | 8 |

Source data of gene expression of hepatic TFs involved in lipid metabolism in SLC mice (Figure 4B).
