# Supplementary material for: Hedgehog signaling is a potent regulator of liver lipid metabolism and reveals a GLI-code associated with steatosis
Source: eLife. 2016 May 17;5:e13308. doi: 10.7554/eLife.13308 (PMC4869931; doi:10.7554/eLife.13308)
Supplement: Figure 5—source data 1. — DOI: http://dx.doi.org/10.7554/eLife.13308.020 [file elife-13308-fig5-data1.docx]

Figure 5 – source data 1

| **figure** | **gene** | **mean SLC-WT** | **SEM SLC-WT** | **n** | **mean SLC-KO** | **SEM SLC-KO** | **p value**  **(t-test)** | **n** |
| --- | --- | --- | --- | --- | --- | --- | --- | --- |
| **5A** | *Gata4* | 1.00 | 0.03 | 24 | 0.56 | 0.03 | < 0.0001*** | 19 |
|  | *Gata6* | 1.00 | 0.05 | 24 | 0.96 | 0.26 | 0.6408 | 19 |
|  | *Nfyb* | 1.00 | 0.15 | 9 | 0.53 | 0.08 | 0.0123* | 10 |
|  | *Nfyg* | 1.00 | 0.19 | 9 | 0.90 | 0.15 | 0.6624 | 10 |
|  | *Lxra* | 1.00 | 0.38 | 12 | 0.861 | 0.40 | 0.7894 | 12 |

| **figure** | **gene** | **mean SLC-WT** | **SEM SLC-WT** | **n** | **mean SLC-KO** | **SEM SLC-KO** | **p value**  **(t-test)** | **n** |
| --- | --- | --- | --- | --- | --- | --- | --- | --- |
| **5B** | *Foxa1* | 1.00 | 0.08 | 12 | 0.98 | 0.04 | 0.8217 | 12 |
|  | *Foxa2* | 1.00 | 0.04 | 12 | 1.02 | 0.05 | 0.7111 | 12 |
|  | *Nr1d1* | 1.00 | 0.13 | 14 | 1.02 | 0.19 | 0.9458 | 14 |
|  | *Nr1d2* | 1.00 | 0.95 | 10 | 0.29 | 0.26 | 0.0087** | 16 |

| **figure** | **gene** | **mean SLC-WT** | **SEM SLC-WT** | **n** | **mean SLC-KO** | **SEM SLC-KO** | **p value**  **(t-test)** | **n** |
| --- | --- | --- | --- | --- | --- | --- | --- | --- |
| **5C** | *Acaca* | 1.00 | 0.22 | 13 | 5.14 | 1.08 | 0.0013** | 14 |
|  | *Acacb* | 1.00 | 0.42 | 6 | 0.79 | 0.22 | 0.6617 | 7 |
|  | *Fasn* | 1.00 | 0.13 | 14 | 1.94 | 0.28 | 0.0028** | 13 |
|  | *Gpam* | 1.00 | 0.18 | 9 | 2.44 | 0.38 | 0.0073** | 15 |
|  | *Elovl6* | 1.00 | 0.34 | 11 | 6.91 | 2.04 | 0.0185* | 14 |
|  | *Elovl3* | 1.00 | 0.30 | 11 | 0.37 | 0.08 | 0.0387* | 13 |

| **figure** | **gene** | **mean SLC-WT** | **SEM SLC-WT** | **n** | **mean SLC-KO** | **SEM SLC-KO** | **p value**  **(t-test)** | **n** |
| --- | --- | --- | --- | --- | --- | --- | --- | --- |
| **5D** | *Aacs* | 1.00 | 0.18 | 14 | 2.98 | 0.56 | 0.0035** | 12 |
|  | *Hmgcr* | 1.00 | 0.27 | 11 | 2.25 | 0.38 | 0.0205* | 16 |
|  | *Lss* | 1.00 | 0.12 | 6 | 1.79 | 0.09 | 0.0003*** | 7 |
|  | *Pnpla3* | 1.00 | 0.59 | 6 | 9.87 | 2.94 | 0.0141* | 6 |

Source data of expression of the hepatic TFs and enzymes involved in lipid metabolism in SLC mice (Figure 5A-D).
