# Supplementary material for: Hedgehog signaling is a potent regulator of liver lipid metabolism and reveals a GLI-code associated with steatosis
Source: eLife. 2016 May 17;5:e13308. doi: 10.7554/eLife.13308 (PMC4869931; doi:10.7554/eLife.13308)
Supplement: Figure 6—source data 1. — DOI: http://dx.doi.org/10.7554/eLife.13308.022 [file elife-13308-fig6-data1.docx]

Figure 6 – source data 1

| **figure** | **analyzes** | **mean SLC-WT**  [µmol/g/h] | **SEM SLC- WT** [µmol/g/h] | **n** | **mean SLC-KO**  [µmol/g/h] | **SEM SLC-KO** [µmol/g/h] | **p value**  **(t-test)** | **n** |
| --- | --- | --- | --- | --- | --- | --- | --- | --- |
| **6B** | fatty biosynthesis | 4.52 | 1.856 | 6 | 3.46 | 1.863 | 0.1821 | 6 |
|  | cholesterol biosynthesis | 17.86 | 2.330 | 6 | 33.71 | 6.850 | 0.0422* | 5 |

| **figure** | **analyzes** | **mean SLC-WT**  [µmol/g/h] | **SEM SLC-**  **WT**  [µmol/g/h] | **n** | **Mean SLC-KO**  [µmol/g/h] | **SEM SLC-KO**  [µmol/g/h] | **p value**  **(t-test)** | **n** |
| --- | --- | --- | --- | --- | --- | --- | --- | --- |
| **6C** | glucose – acetate control | 0.007 | 0.001 | 6 | 0.010 | 0.001 | 0.361 | 6 |
|  | glucose – acetate (glucose + insulin) | 320.85 | 78.62 | 6 | 585.28 | 42.76 | 0.0144* | 6 |

| **figure** | **analyzes** | **mean SLC-WT**  [mg clycogen/mg DNA] | **SEM SLC-**  **WT**  [mg clycogen/mg DNA] | **n** | **mean SLC-KO**  [mg clycogen / mg DNA] | **SEM SLC-KO**  [mg clycogen/mg DNA] | **p value**  **(t-test)** | **n** |
| --- | --- | --- | --- | --- | --- | --- | --- | --- |
| **6D** | glycogen content | 1.835 | 0.450 | 5 | 1.9932 | 0.4970 | 0.2721 | 5 |

| **figure** | **analyzes** | **mean SLC-WT**  [µmol/g/h] | **SEM SLC-**  **WT**  [µmol/g/h] | **n** | **mean SLC-KO**  [µmol/g/h] | **SEM SLC-KO**  [µmol/g/h] | **p value**  **(t-test)** | **n** |
| --- | --- | --- | --- | --- | --- | --- | --- | --- |
| **6E** | glykolysis control | 3.930 | 0.839 | 5 | 4.5703 | 0.9163 | 0.6205 | 5 |
|  | glycolysis (glucose + insulin) | 149637.617 | 30285.416 | 5 | 161749.830 | 22971.624 | 0.7582 | 5 |

| **figure** | **analyzes** | **mean SLC-WT**  [RLU] | **SEM SLC-**  **WT**  [RLU] | **n** | **mean SLC-KO**  [RLU] | **SEM SLC-KO**  [RLU] | **p value**  **(t-test)** | **n** |
| --- | --- | --- | --- | --- | --- | --- | --- | --- |
| **6G** | ATP content | 899.47 | 308.95 | 5 | 396.96 | 52.94 | 0.0286* | 12 |

| **figure** | **gene** | **mean SLC-WT** | **SEM SLC-WT** | **n** | **mean SLC-KO** | **SEM SLC-KO** | **p value**  **(t-test)** | **n** |
| --- | --- | --- | --- | --- | --- | --- | --- | --- |
| **6H** | *Acox1* | 1.00 | 0.17 | 6 | 0.88 | 0.10 | 0.5682 | 6 |
|  | *Cpt1a* | 1.00 | 0.28 | 6 | 0.82 | 0.21 | 0.625 | 6 |
|  | *Cpt2* | 1.00 | 0.21 | 6 | 0.77 | 0.32 | 0.5596 | 6 |
|  | *Acadvl* | 1.00 | 0.12 | 6 | 0.78 | 0.05 | 0.1238 | 6 |

| **figure** | **gene** | **mean SLC-WT** | **SEM SLC-WT** | **n** | **mean SLC-KO** | **SEM SLC-KO** | **p value**  **(t-test)** | **n** |
| --- | --- | --- | --- | --- | --- | --- | --- | --- |
| **6I** | *Slc25a1* | 1.00 | 0.12 | 6 | 1.00 | 0.08 | 0.9768 | 6 |
|  | *Slc25a5* | 1.00 | 0.11 | 6 | 0.63 | 0.07 | 0.0143* | 6 |
|  | *Slc25a20* | 1.00 | 0.19 | 6 | 1.64 | 0.31 | 0.1054 | 6 |

| **figure** | **serum parameter** | **mean SLC-**  **WT**  [µmol/l] | **SEM**  **SLC-WT**  [µmol/l] | **n** | **mean SLC-KO**  [µmol/l] | **SEM SLC-KO**  [µmol/l] | **p value**  **(t-test)** | **n** |
| --- | --- | --- | --- | --- | --- | --- | --- | --- |
| **6J** | *acetoacetate* | 32.96 | 8.71 | 5 | 25.04 | 7.47 | 0.5097 | 5 |
|  | *3-hydroxybutyrate* | 74.24 | 8.12 | 5 | 106.40 | 13.05 | 0.0698 | 5 |
|  | *total ketone bodies* | 107.20 | 12.57 | 5 | 131.44 | 7.67 | 0.1383 | 5 |

Source data of expression of genes and proteins involved in lipid and mitochondrial energy metabolism in SLC mice (Figure 6B-J)
