# Supplementary material for: Hedgehog signaling is a potent regulator of liver lipid metabolism and reveals a GLI-code associated with steatosis
Source: eLife. 2016 May 17;5:e13308. doi: 10.7554/eLife.13308 (PMC4869931; doi:10.7554/eLife.13308)
Supplement: Figure 7—source data 1. — DOI: http://dx.doi.org/10.7554/eLife.13308.025 [file elife-13308-fig7-data1.docx]

Figure 7 – source data 1

| **figure** | **gene** | **siRNA** | **mean** | **SEM** | **p value**  **(paired t-test)** | **n** |
| --- | --- | --- | --- | --- | --- | --- |
| **7A** | *Ppara* | nonsense | 1.0 | 0.37 - 0.58 |  | 4 - 7 |
|  |  | *Gli1* | 1.09 | 0.35 | 0.6201 | 4 |
|  |  | *Gli2* | 1.11 | 0.34 | 0.6266 | 4 |
|  |  | *Gli3* | 5.51 | 1.91 | 0.0428* | 7 |
|  | *Pparg* | nonsense | 1.0 | 0.07 - 0.17 |  | 9 - 13 |
|  |  | *Gli1* | 0.83 | 0.15 | 0.5067 | 9 |
|  |  | *Gli2* | 0.81 | 0.08 | 0.0801 | 9 |
|  |  | *Gli3* | 3.59 | 1.44 | 0.0684 | 13 |
|  | *Srebf1* | nonsense | 1.0 | 0.18 - 0.27 |  | 4 - 7 |
|  |  | *Gli1* | 2,17 | 0.09 | 0.0016** | 6 |
|  |  | *Gli2* | 1.17 | 0.12 | 0.3877 | 4 |
|  |  | *Gli3* | 1.90 | 0.28 | 0.0338* | 7 |
|  | *Srebf2* | nonsense | 1.0 | 0.08 – 0.32 |  | 5 - 9 |
|  |  | *Gli1* | 1.01 | 0.13 | 0.947 | 5 |
|  |  | *Gli2* | 1.07 | 0.06 | 0.7191 | 9 |
|  |  | *Gli3* | 1.10 | 0.20 | 0.8525 | 9 |
|  | *Chrebp* | nonsense | 1.0 | 0.21 – 0.29 |  | 4 - 5 |
|  |  | *Gli1* | 0.96 | 0.23 | 0.9333 | 4 |
|  |  | *Gli2* | 0.85 | 0.19 | 0.4293 | 5 |
|  |  | *Gli3* | 0.83 | 0.08 | 0.1162 | 5 |

| **figure** | **analyzes** | **siRNA** | **mean** | **SEM** | **p value**  **(paired t-test)** | **n** |
| --- | --- | --- | --- | --- | --- | --- |
| **7C** | fat red quantify-cation | nonsense | 1.00. | 0.9 – 0.16 |  | 4 - 6 |
|  |  | *Gli1* | 1.24 | 0.15 | 0.236 | 4 |
|  |  | *Gli2* | 1.03 | 0.09 | 0.639 | 6 |
|  |  | *Gli3* | 2.06 | 0.23 | 0.0018** | 6 |

Source data of the influence of siRNA-mediated knockdown of *Gli1*, *Gli2* and *Gli3* on expression of genes of lipid metabolism (Figure 7A, C).
