# Supplementary material for: Hedgehog signaling is a potent regulator of liver lipid metabolism and reveals a GLI-code associated with steatosis
Source: eLife. 2016 May 17;5:e13308. doi: 10.7554/eLife.13308 (PMC4869931; doi:10.7554/eLife.13308)
Supplement: Figure 8—source data 1. — DOI: http://dx.doi.org/10.7554/eLife.13308.029 [file elife-13308-fig8-data1.docx]

Figure 8 – source data 1

| **figure** | **gene** | **siRNA** | **mean** | **SEM** | **p value**  **(paired t-test)** | **n** |
| --- | --- | --- | --- | --- | --- | --- |
| **8A** | *Sufu* | nonsense | 1.00 | 1.03 |  | 9 |
|  |  | Sufu | 0.12 | 0.03 | 0.0028** | 9 |
|  | *Fu* | nonsense | 1.00 | 0.27 |  | 8 |
|  |  | Sufu | 1.35 | 0.21 | 0.2358 | 8 |

| **figure** | **gene** | **siRNA** | **mean** | **SEM** | **p value**  **(paired t-test)** | **n** |
| --- | --- | --- | --- | --- | --- | --- |
| **8B** | *Gli1* | nonsense | 1.00 | 0.23 |  | 11 |
|  |  | Sufu | 2.47 | 0.67 | 0.1385 | 11 |
|  | *Gli2* | nonsense | 1.00 | 0.28 |  | 11 |
|  |  | Sufu | 3.37 | 0.70 | 0.0016** | 11 |
|  | *Gli3* | nonsense | 1.00 | 0.25 |  | 11 |
|  |  | Sufu | 3.27 | 0.75 | 0.0269* | 11 |

| **figure** | **gene** | **siRNA** | **mean** | **SEM** | **p value**  **(paired t-test)** | **n** |
| --- | --- | --- | --- | --- | --- | --- |
| **8C** | *Srebf1* | nonsense | 1.00 | 0.53 |  | 11 |
|  |  | Sufu | 0.76 | 0.06 | 0.0097** | 11 |
|  | *Srebf2* | nonsense | 1.00 | 0.11 |  | 11 |
|  |  | Sufu | 0.85 | 0.13 | 0.1435 | 11 |
|  | *Pparg* | nonsense | 1.00 | 0.33 |  | 10 |
|  |  | Sufu | 0.95 | 0.28 | 0.5277 | 10 |
|  | *Elovl3* | nonsense | 1.00 | 0.10 |  | 9 |
|  |  | Sufu | 0.88 | 0.15 | 0.4405 | 9 |
|  | *Elovl6* | nonsense | 1.00 | 0.09 |  | 11 |
|  |  | Sufu | 0.89 | 0.12 | 0.2575 | 11 |

| **figure** | **analyzes** | **siRNA** | **mean** | **SEM** | **p value**  **(paired t-test)** | **n** |
| --- | --- | --- | --- | --- | --- | --- |
| **8E** | fat red quantification | nonsense | 1.00 | 0.30 |  | 7 |
|  |  | Sufu | 0.54 | 0.09 | 0.0399* | 7 |

Data source of the influence of siRNA-mediated knockdown of *Sufu* on lipid metabolism in vitro (Figure 8 A-C, D).
