# Supplementary material for: Hedgehog signaling is a potent regulator of liver lipid metabolism and reveals a GLI-code associated with steatosis
Source: eLife. 2016 May 17;5:e13308. doi: 10.7554/eLife.13308 (PMC4869931; doi:10.7554/eLife.13308)
Supplement: Figure 9—source data 1. — DOI: http://dx.doi.org/10.7554/eLife.13308.031 [file elife-13308-fig9-data1.docx]

Figure 9 – source data 1

| **figure** | **transfection** | **gene** | **mean** | **SEM** | **n** |
| --- | --- | --- | --- | --- | --- |
| **9A** | MOCK | *Gli1* | 1.00 | 0.62 | 3 (pooled) |
|  | GLI1 | *Gli1* | 7596.53 | 3684.47 | 3 (pooled) |
|  | GLI2 | *Gli1* | 19.60 | 15.17 | 3 (pooled) |
|  | GLI3 | *Gli1* | 10.97 | 6.45 | 3 (pooled) |
|  | GLI1/Gli3 | *Gli1* | 1379.31 | 660.64 | 3 (pooled) |

| **figure** | **transfection** | **gene** | **mean** | **SEM** | **n** |
| --- | --- | --- | --- | --- | --- |
| **9B** | MOCK | *Gli2* | 1.00 | 0.03 | 3 (pooled) |
|  | GLI1 | *Gli2* | 32.50 | 22.07 | 3 (pooled) |
|  | GLI2 | *Gli2* | 2787.27 | 1627.91 | 3 (pooled) |
|  | GLI3 | *Gli2* | 17.89 | 13.26 | 3 (pooled) |
|  | GLI1/Gli3 | *Gli2* | 1.01 | 0.17 | 3 (pooled) |

| **figure** | **transfection** | **gene** | **mean** | **SEM** | **n** |
| --- | --- | --- | --- | --- | --- |
| **9C** | MOCK | *Gli3* | 1.00 | 0.15 | 3 (pooled) |
|  | GLI1 | *Gli3* | 6.44 | 2.75 | 3 (pooled) |
|  | GLI2 | *Gli3* | 18.06 | 9.34 | 3 (pooled) |
|  | GLI3 | *Gli3* | 1991.16 | 985.08 | 3 (pooled) |
|  | GLI1/Gli3 | *Gli3* | 767.79 | 382.66 | 3 (pooled) |

| **figure** | **transfection** | **analyzes** | **mean** | **SEM** | **p value**  **(paired t-test** | **n** |
| --- | --- | --- | --- | --- | --- | --- |
| **9D** | MOCK | fat red quantification | 1.0 | 0.08 |  | 3 (pooled) |
|  | GLI1 | fat red quantification | 1.0 | 0.11 | 0.7735 | 3 (pooled) |
|  | GLI2 | fat red quantification | 1.11 | 0.14 | 0.2621 | 3 (pooled) |
|  | GLI3 | fat red quantification | 1.11 | 0.23 | 0.4728 | 3 (pooled) |
|  | GLI1/Gli3 | fat red quantification | 0.54 | 0.14 | 0.046* | 3 (pooled) |

Source data of the influence of GLI1, GLI2 and GLI3 overexpression on lipid content in hepatocytes of ob/ob mice (Figure A-D).
