# Supplementary material for: Hedgehog signaling is a potent regulator of liver lipid metabolism and reveals a GLI-code associated with steatosis
Source: eLife. 2016 May 17;5:e13308. doi: 10.7554/eLife.13308 (PMC4869931; doi:10.7554/eLife.13308)
Supplement: Supplementary file 1. (A) — (B) Primers for qRT-PCR on murine mRNA. (C) Primers for siRNA mediated knockdown of Sufu. (D) Primers for ChIP (E) Primers for human Gli factors DOI: http://dx.doi.org/10.7554/eLife.13308.033 [file elife-13308-supp1.docx]

Supplementary file 1

**Supplementary file 1A**

**Primers for genotyping the SLC mice.**

| **gene** | **primer** |
| --- | --- |
| *Smo* WT | forward ccactgcgagcctttgcgctac  reverse cccatcacctccgcgtcgca |
| *Smo* flx. | forward atggccgctggccgccccgtg  reverse ggcgctaccggtggatgtgg |
| *Smo* rec. | forward ggcctgcgctgctcaacatgg  reverse ccatcacgtcgaactcctggc |
| *rtetR* | forward ccatgtctagactggacaaga  reverse ctccaggccacatatgattag |
| Cre recombinase | forward tcgctgcattaccggtcgatgc  reverse ccatgagtgaacgaacctggtcg |

**Supplementary file 1B**

**Primers for qRT-PCR on murine mRNA.**

| **gene** | **primer** |
| --- | --- |
| *Aacs* | forward aggagcgggtggtcctgttc  reverse tcacggccacctccactttc |
| *Acaca* | forward cccaaaggcaaagtgaagac  reverse atccgcctccaaaaagaact |
| *Acacb* | forward tccacttcctggagctgaac  reverse gctcaagggggtttcaaaag |
| *Acadvl* | forward ggctgctttctgcctaacag  reverse cgtggctgcatctttaattg |
| *Acox1* | forward gcccaactgtgacttccatc  reverse gccaggactatcgcatgatt |
| *Actb* | Forward catccgtaaagacctctatgccaac  reverse atggagccaccgatccaca |
| *Chrebp1* | forward acatcagcgctttgaccag  reverse taaaggtcggatgaggat |
| *Cpt1a* | forward gctgtcaaagataccgtgagc  reverse tctccctccttcatcagtgg |
| *Cpt2* | forward aagcctctcttgaatgacagc  reverse ccaatgccgttctcaaaatc |
| *Elovl3* | forward ctgttgctcatcgttgttgg  reverse atccgtgtagatggcaaagc |
| *Elovl6* | forward tctgggcttatgcatttgtg  reverse acaggagcacagtgatgtgg |
| *Fasn* | forward tagagggagccagagagacg  reverse ttggcccagaactcctgtag |
| *Foxa1* | forward gaacagctactacgcggaca  reverse cggagttcatgttgctgaca |
| *Foxa2* | forward tacccagggggctatggt  reverse cccgctttgttcgtgact |
| *Fu* | forward tgcctctcagccttcttagg  reverse taagagcgccccatacca |
| *Gli1* | forward cagggaagagagcagactgac  reverse cgctgctgcaagaggact |
| *Gli2* | forward actttctccacaccctgctg  reverse ggctgcgaggctaaagagtc |
| *Gli3* | forward ctggcttgattgttcacgag  reverse cagccctcatgctcacagac |
| *Gpam* | forward tcctggccttgcagaacagc  reverse gcaacgttcctttccgtcctg |
| *Hhip* | forward ctacttgggccagatggaag  reverse ctccaagtaaggctccttgaac |
| *Hmgcr* | forward tgattggagttggcaccat  reverse tggccaacactgacatgc |
| *Ihh* | forward gctcacccccaactacaatc  reverse gcggccctcatagtgtaaag |
| *Lss* | forward ttccatgaacacttcccaga  reverse aggccttgattgagggtctc |
| *Lxra* | forward aagggagcacgctatgtctg  reverse cttcttgccgcttcagtttc |
| *Nfyb* | forward gatccagccccatgatgata  reverse tgaaactttcttttgaaccatttg |
| *Nfyg* | forward gctaccaatgcccaacagat  reverse tccatctgtgaactggctga |
| *Ppara* | forward cgtacggcaatggctttatc  reverse tcatctggatggttgctctg |
| *Pparb/d* | forward cagcctcaacatggaatgtc  reverse cagatccgatcgcacttctc |
| *Pparg* | forward atggaagaccactcgcattc  reverse gctttatccccacagactcg |
| *Pnpla3* | forward tgggagagctgtgctatcaa  reverse gctgtggcccgttacaga |
| *Ptch1* | forward cctcctttacggtggacaaac  reverse atcaactcctcctgccaatg |
| *Ptch2* | forward ccgagtggctgtaattgagac  reverse ctggaggtgcaagtcaagtg |
| *Nr1d1* | forward acgaccctggactccaataa  reverse ccattggagctgtcactgtaga |
| *Nr1d2* | forward acagaaatagttacctgtgcaacact  reverse gacttgctcataggacacacca |
| *Slc25a1* | forward ggcacacaaataccggaac  reverse aatacgatggccacatccag |
| *Slc25a5* | forward gatgccgctgtgtccttc  reverse tatctgccgtgatttgcttg |
| *Slc25a20* | forward aaatctccagaggatgaacttagc  reverse cctgtggtgaacacaccagata |
| *Shh* | forward tccaaagctcacatccactg  reverse ctccgggacgtaagtccttc |
| *Smo* | forward gcaagctcgtgctctggt  reverse gggcatgtagacagcacaca |
| *Srebf1* | forward aagcgctaccggtcttctatc  reverse tgtgcacttcgtagggtcag |
| *Srebf2* | forward agccctacccgtacacacc  reverse gatggcagtagctcgctctc |
| *Sufu* | forward cttccagtcagagaacacct  reversettgggctgaatgtaactc |

**Supplementary file 1C**

**Primers for siRNA mediated knockdown of *Sufu*.**

| **gene** | **primer** |
| --- | --- |
| *Sufu* | CCCUUGGACUAUGUUAGCAUGUACA  UGUACAUGCUAACAUAGUCCAAGGG |

**Supplementary file 1D**

**Primers for human Gli factors**

| **Gene** | **Sequence** |
| --- | --- |
| *Gli1* | forward agtctgagctggacatgctg  reverse acaggggatcctgtatgcct |
| *Gli2* | forward agtcactcaagaattcctgctca  reverse gttttccaggatggagccactt |
| *Gli3* | forward gctccacgaccactgaaaag  reverse tgtggctgcatagtgattgc |

**Supplementary file 1E**

**Primers for ChIP**

| **Gene** | **Sequence** |
| --- | --- |
| *Ppara* | forward gctttgaagatcagcagaggg  reverse ccaggctcgtctatgtttacc |
| *Fasn* | forward tgggcctctttaagtcatgc  reverse cacctgcaaagtatgtcccc |
| *Srebf1* | forward gcagaacctacgagtcaacc  reverse ggttgggaggtgaaagcag |
| *beta Actin* | forward catccgtaaagacctctatgccaac  reverse atggagccaccgatccaca |
| *Rpl30* | SimpleChiP Mouse RPL30 Primer #7015 (Cell signaling) |
